# *Piper betle* extract and its application in bovine teat dipping solution inhibit and eliminate biofilms in bovine mastitis-inducing staphylococci

**DOI:** 10.14202/vetworld.2023.2135-2142

**Published:** 2023-10-18

**Authors:** Paparwee Sungkatavat, Haemarat Khongkhai, Wilasinee Kanchana, Phirabhat Saengsawarng, Suthinee Sangkanu, Veeranoot Nissapatorn, Maria de Lourdes Pereira, Julalak Chorachoo Ontong, Watcharapong Mitsuwan

**Affiliations:** 1 Akkhraratchakumari Veterinary College, Walailak University, Nakhon Si Thammarat, 80160, Thailand; 2 One Health Research Center, Walailak University, Nakhon Si Thammarat, 80160, Thailand; 3 Division of Biological Science, Faculty of Sciences, Prince of Songkla University, Hat Yai, Songkhla 90112, Thailand; 4 School of Allied Health Sciences, Southeast Asia Water Team, World Union for Herbal Drug Discovery, and Research Excellence Center for Innovation and Health Products, Walailak University, Nakhon Si Thammarat, Thailand; 5 CICECO-Aveiro Institute of Materials and Department of Medical Sciences, University of Aveiro, 3810-193 Aveiro, Portugal; 6 Cosmetic Technology and Dietary Supplement Products Program, Faculty of Agro and Bio Industry, Thaksin University, Phatthalung, Thailand; 7 Center of Excellence in Innovation of Essential Oil and Bioactive Compounds, Walailak University, Nakhon Si Thammarat, 80160, Thailand

**Keywords:** antibacterial activity, biofilms, bovine teat dipping solution, *Piper betle* extract, staphylococci

## Abstract

**Background and Aim:**

Staphylococci, including *Staphylococcus aureus*, *Staphylococcus* *chromogenes*, and *Staphylococcus haemolyticus*, are significant bacteria that induce bovine mastitis, primarily because they can form biofilms in bovine teat canals. This study aimed to investigate the efficacy of *Piper betle* extract and a bovine teat dipping solution containing *P. betle* extract (BSP) against these mastitis-causing staphylococci.

**Materials and Methods:**

BSP was prepared using *P. betle* extract as the bioactive compound. The antibacterial activity of the plant extract and BSP against the pathogens was investigated using a broth microdilution method. The activity of the extract and BSP against the pathogen biofilms was also determined. A stability test was performed to observe the pH, color, turbidity, homogeneity, precipitation, and separation of BSP stored at 4°C and 25°C for up to 4 weeks.

**Results:**

The extract exhibited potent antibacterial activity against *S. aureus* and *S. haemolyticus*, with similar values for minimum inhibitory concentration (MIC) and minimum bactericidal concentration (MBC) ranging from 0.03 mg/mL to 0.125 mg/mL. The MIC and MBC values of the extract against *S. chromogenes* were 0.5–1 mg/mL and 0.5–2 mg/mL, respectively. Moreover, BSP exhibited MIC and MBC values of 12.5–50 v/v against all tested staphylococci isolates. When used at 1/2 and 1/4 × MIC, the extract and BSP significantly inhibited the formation of staphylococcal biofilms (p < 0.05) in the tested strains. The results indicated that treatment with 1/2 × MIC of the extract and BSP resulted in biofilm inhibition ranging from 30%–66% and 19%–39%, respectively. Furthermore, the extract at 16 × MIC eliminated 54%–86% of established mature isolate biofilms, whereas BSP removed 41%–61% of mature biofilm viability. Storage of BSP at 4°C did not change the factors associated with stability from the 1^st^ to 4^th^ week.

**Conclusion:**

These findings suggest that BSP may exhibit potential medicinal benefits in inhibiting the growth and biofilm formation of mastitis-inducing staphylococci in bovines.

## Introduction

Bovine mastitis is a common disease in dairy cattle, causing significant economic losses due to reduced milk production and poor milk quality [1]. In Thailand, an increased somatic cell count (SCC) in milk can reduce its commercial value, causing an average economic loss of $557 for farms experiencing high SCC over 3 months [2]. Bovine mastitis is a mammary gland inflammation caused by physical trauma or infections from different bacteria, particularly *Staphylococcus* spp., a prevalent bacterial pathogen responsible for mastitis in Thailand [1]. *Staphylococcus aureus*, a coagulase-positive *Staphylococcus*, is primarily responsible for clinical bovine mastitis, whereas coagulase-negative *Staphylococcus chromogenes* and *Staphylococcus haemolyticus* are causative agents of subclinical bovine mastitis, characterized by no clinical signs. However, they are detectable in the milk of infected dairy cows. These bacteria adhere to and invade bovine mammary epithelial cells, forming a biofilm, and persistently infecting the udder [3].

Despite standard antibiotic treatment, certain cases of mastitis remain unresolved, causing treatment failure and increased antibiotic use within the dairy industry [4]. In addition, chronic infections caused by biofilms, primarily produced by *Staphylococcus* spp., are challenging to eliminate with antibiotics [4, 5]. This reliance on antibiotics for mastitis treatment has negative consequences relating to antibiotic residues in the human food chain and the potential transmission of antibiotic-resistant bacterial strains [6]. Importantly, environmental pathogens can cause bovine mammary parenchyma infection after milking, resulting from udder injury caused by the milking machine. Therefore, to address antibiotic resistance, a post-milking natural-based teat disinfectant could help prevent and control bovine mastitis.

Natural substances, including medicinal plants, can be considered as alternative remedies against pathogenic hazards. Medicinal plants have been adopted in many countries as substitutes for wound healing, diarrhea treatment, tuberculosis, and cancer management [7]. Recently, *Knema retusa*, a native plant in Southeast Asia, exhibited anti-staphylococcal and antibiofilm activities against *S. aureus* and *S. haemolyticus* isolated from mastitis-affected dairy cows [1]. This study focused on the extract of *Piper betle* leaf, which belongs to the *Piperaceae* family and is native to South and Southeast Asia. *Piper* *betle* leaf has been widely used as an alternative treatment because of its antimicrobial properties [8]. It contains different chemical constituents, including hydroxychavicol, eugenol, chavicol, and other phenolic compounds having potent antifungal and antibacterial properties [8, 9]. Recently, the activities of *P. betle* extract against adhesion and biofilm formation of avian pathogenic *Escherichia coli* (APEC) have been reported [10].

Therefore, this study aimed to investigate the antibacterial and antibiofilm effectiveness of *P. betle* extract and a bovine teat dipping solution containing *P. betle* extract (BSP) against bovine mastitis-inducing staphylococci, namely, *S. aureus*, *S. chromogenes*, and *S. haemolyticus*. Furthermore, BSP characterization and stability were evaluated.

## Materials and Methods

### Ethical approval

This article does not contain any studies performed by any of the authors with human or animal participants.

### Study period and location

The study was conducted from January 2022 to April 2023. Samples of *P. betle* leaves were collected from Phatthalung province, South Thailand. All experiments were performed at the bacterial laboratory, Walailak University, Nakhon Si Thammarat, Thailand.

### Plant extract preparation

Mature leaves of *P. betle*, collected from Phatthalung province, Thailand, were washed and air-dried in an oven at 40°C for 3 days. Subsequently, the betel leaves were cut into small pieces and powdered using a dry blender. Then, 50 g of the dried plant powder was soaked at room temperature with 200 mL of 95% ethanol for 7 days [11]. The solution was filtered using filter paper No. 1 (Whatman International Ltd., Maidstone, UK). The sample was then evaporated under reduced pressure using a vacuum evaporator (Buchi Labortechnik AG, Flawil, Switzerland) to obtain the extract. The plant extract was dissolved in 100% dimethyl sulfoxide (DMSO) and/or absolute ethanol as stock solutions (200 mg/mL). For positive control treatment, vancomycin was dissolved in 100% DMSO. The extract and vancomycin were stored at 20°C until further use.

### Preparation and stability test of the BSP

Bovine teat dipping solution containing *P. betle* extract was prepared using *P. betle* extract as the bioactive compound, following the method described by Zhang *et al*. [12], with modifications. For BSP preparation, the extract was dissolved in absolute ethanol at 200 mg/mL. Xanthan gum (2.5 g) was dissolved in 500 g distilled water and then heated at 150°C for 1 h to ensure complete dissolution. Next, 25 g glycerin and 25 g propylene glycol were added and homogenized. The solution (195 mL) was aliquoted into two sterile bottles. Next, 5 mL *P. betle* extract was added to ethanol and mixed, and the gel base solution was prepared by adding 5 mL absolute ethanol. A stability test was conducted to observe the BSP pH, color, turbidity, homogeneity, precipitation, and separation stored at 4°C and 25°C for 4 weeks.

### Preliminary screening of the *P. betle* extract against staphylococci

A paper disk diffusion method was used to test the activity of the extract against clinical staphylococcal isolates, including *S. aureus*, *S. chromogenes*, and *S. haemolyticus*. Briefly, 12.5 μL of the stock solution at 200 mg/mL in 100% DMSO was loaded onto sterile filter paper disks 6 mm in diameter, resulting in a concentration of 2.5 mg/disk. To prepare the bacterial culture, 3–5 colonies of each bacterium were cultured in Mueller–Hinton broth (MHB) (Difco, Claix, France) for 3–5 h at 37°C. The bacterial suspension was adjusted to match the turbidity of the McFarland No. 0.5 standard and then swabbed on Mueller–Hinton agar (Difco) plates. Subsequently, extract containing disks were placed on bacterial culture plates and incubated at 37°C for 18 h. Ampicillin and clindamycin (Oxoid, Hampshire, UK) were positive controls, and a disk containing 100% DMSO was the negative control. The zone of inhibition was measured using a Vernier caliper and recorded as previously described by Chuprom *et al*. [1]. The results are presented as mean ± standard deviation (SD) calculated in triplicate.

### Minimum inhibitory concentration (MIC) and minimum bactericidal concentration (MBC) of the extract and BSP against pathogens

The MIC and minimum MBC values of the extract and BSP against the clinical isolates of staphylococci were investigated using a broth microdilution assay, following the method described by Mitsuwan *et al*. [13]. Briefly, the extract and BSP were diluted in a 96-well plate containing MHB to final concentrations ranging from 2.0 mg/mL to 0.125 mg/mL and 100 v/v to 3.125 v/v, respectively. The bacterial culture of each tested isolate was prepared as described earlier. Subsequently, 100 µL of the bacterial suspension (1 × 10^6^ colony-forming unit [CFU]/mL) was added to the microtiter plate containing the antimicrobial agents. Vancomycin was used as the positive control. For the MIC and MBC determination of the extract, 1% DMSO was used as the negative control, whereas for BSP, 1% ethanol and the gel base solution served as the negative control. The microtiter plates were incubated at 37°C for 18 h. Subsequently, resazurin (0.03%) (Thermo Fisher Scientific, Lancashire, UK) was added to the wells for MIC determination by observing color changes. The MIC was defined as the lowest concentration completely inhibiting bacterial growth, indicated by blue. For MBC detection, the samples that showed blue were cultured on trypticase soy agar plates and incubated at 37°C for 24 h. The experiment was repeated thrice data authentication.

### Effects of *P. betle* extract and BSP against staphylococcal biofilm formation

The activity of the extract and BSP against biofilm formation of clinical staphylococcal isolates was evaluated using the crystal violet assay, following the method described by Mitsuwan *et al*. [14]. Initially, 3–5 isolate colonies were cultured in trypticase soy broth (Difco) supplemented with 0.5% glucose (Sigma, USA) and incubated at 37°C for 18–24 h. Then, the bacterial suspension (2 × 10^6^ CFU/mL) was incubated with sub-MICs of the extract or BSP at 37°C for 24 h. For comparison, 1% DMSO and the gel base solution were used as controls for the extract and BSP, respectively. The effects of the extract on bacterial growth after incubation were evaluated by measuring the optical density (OD) at 600 nm. The wells containing the biofilms were washed using phosphate-buffered saline (PBS), air-dried, and stained with 200-µL 0.1% (w/v) crystal violet for 30 min. Excess dye was removed by washing twice with distilled water. The samples were air-dried, dissolved in DMSO, and measured at 570 nm. The relative percentage of biofilm formation was calculated as (mean OD_570_ of treated well/mean OD_570_ of control well) × 100.

### Effects of *P. betle* extract and BSP on established biofilms

The activity of the extract and BSP in eliminating established staphylococcal biofilms was determined as described by Mitsuwan *et al* [14]. Initially, 200-µL bacterial culture, as described earlier, was added to a 96-well microtiter plate and incubated at 37°C for 2 days to establish early/young biofilms and 7 days to establish mature biofilms. The planktonic cells were removed every 48 h, and fresh medium was added. After incubation, the medium was removed, and the wells were rinsed with PBS twice. To evaluate the activity of the *P. betle* extract, the established biofilms were treated with the extract at concentrations ranging from 4 × MIC to 16 × MIC. For BSP, 50%–100% v/v concentrations were applied to treat the established biofilm. Dimethyl sulfoxide (1%) served as the negative control for the extract, whereas 1% ethanol and the gel base solution served as negative controls for BSP. Then, the medium was substituted with 200-µL PBS containing 10-µL - 3-(4,5-dimethylthiazol-2-yl)-2,5-diphenyltetrazolium bromide (MTT) (5 mg/mL; Sigma-Aldrich, Missouri, USA) and further incubated at 37°C for 2 h. 3-(4,5-Dimethylthiazol-2-yl)-2,5-diphenyltetrazolium bromide is digested by the dehydrogenase enzyme in living bacterial cells within the biofilm, producing insoluble purple formazan. The samples were dissolved in DMSO, and the absorbance was measured at 570 nm. The relative percentage of biofilm formation was calculated as (mean OD_570_ of treated well/mean OD_570_ of control well) × 100.

### Statistical analysis

The experiments were performed in triplicate, presenting the data as mean ± SD. Statistical analysis was conducted using one-way analysis of variance (Statistical Package for the Social Sciences Inc., version 15, Chicago, IL, USA). Comparisons between means were performed using a *post hoc* test. p < 0.05 was considered statistically significant.

## Results

### Characterization and stability of the BSP

**Figure-1:**
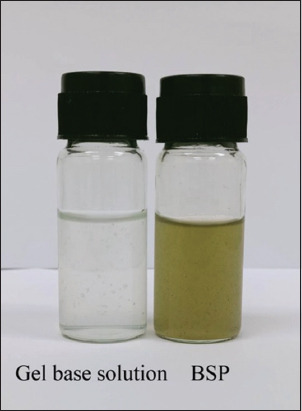
Physical appearance of Bovine teat dipping solution containing *Piper betle* extract and gel base solution.

This study used BSP as an alternative agent against bovine mastitis-inducing staphylococci. The solution exhibited a greenish-brown color with translucent turbidity (Figure-1). BSP exhibited homogeneity and no signs of precipitation or separation.

To assess BSP stability, various factors associated with the solution’s stability were evaluated for 4 weeks (Table-1). During storage at 4°C, the stability-related factors remained unchanged until the 4^th^ week. However, the pH values slightly decreased at the 4^th^ week. However, when stored at 25°C, the BSP color changed to dark greenish-brown starting from the 1^st^ week. In addition, pH values decreased at the 4^th^ week compared with week 0. Nevertheless, BSP turbidity, homogeneity, precipitation, and separation exhibited no noticeable changes.

**Table-1:** Stability of bovine teat dipping solution containing *Piper betle* extract during the time storage.

Storage	Stability	Week				

		0	1	2	3	4
4°C	Color	Greenish brown	Greenish brown	Greenish brown	Greenish brown	Greenish brown
	Turbidity	Translucent	Translucent	Translucent	Translucent	Translucent
	pH	6.84 ± 0.03	6.78 ± 0.02	6.68 ± 0.04	6.65 ± 1.09	6.64 ± 0.02
	Homogeneity	Yes	Yes	Yes	Yes	Yes
	Precipitation	No	No	No	No	No
	Separation	No	No	No	No	No
25°C	Color	Greenish brown	Greenish brown +	Greenish brown +	Greenish brown +	Greenish brown +
	Turbidity	Translucent	Translucent	Translucent	Translucent	Translucent
	pH	6.84 ± 0.03	6.43 ± 0.28	6.55 ± 0.03	6.21 ± 0.02	6.21 ± 0.02
	Homogeneity	Yes	Yes	Yes	Yes	Yes
	Precipitation	No	No	No	No	No
	Separation	No	No	No	No	No

### Preliminary antibacterial activity of *P. betle* extract against staphylococci

**Figure-2:**
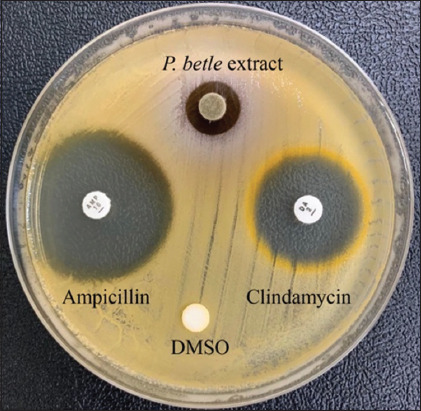
The activity of *Piper betle* extract against representatively isolate staphylococci, *Staphylococcus chromogenes* WU211024, as measured by disk diffusion assay. Ampicillin and clindamycin were used as positive controls, while dimethyl sulfoxide was included as a negative control. Inhibitory activity was presented as zones of inhibition.

The preliminary antibacterial activity of *P. betle* extract against clinical isolates of bovine mastitis-inducing staphylococci was assessed (Figure-2). The extract exhibited inhibition zones against staphylococci, including *S. aureus*, *S. chromogenes*, and *S. haemolyticus*, ranging from 17.67 ± 0.47–22.00 ± 0.82, 13.33 ± 0.47–20.33 ± 0.47, and 20.00 ± 0.00–25.33 ± 0.47-mm, respectively (Table-2). The inhibition zones of the extract against the reference strain were within the same range as those observed for the tested clinical isolates.

**Table-2:** Antibacterial activity of the *Piper betle* leaf extract against *Staphylococcus aureus*, *Staphylococcus chromogenes*, and *Staphylococcus haemolyticus*.

Clinical isolates	Inhibition zone (mm)	Antibiotics (% susceptibility)	

		Ampicillin	Clindamycin
*Staphylococcus aureus*(n = 8)	17.67 ± 0.47–22.00 ± 0.82	60	100
*Staphylococcus chromogenes*(n = 8)	13.33 ± 0.47–20.33 ± 0.47	60	100
*Staphylococcus haemolyticus*(n = 8)	20.00 ± 0.00–25.33 ± 0.47	100	100
*Staphylococcus aureus*ATCC25923	17.00 ± 0.00	Sensitive	Sensitive

### Minimum inhibitory concentration and MBC values of the extract and BSP against clinical isolates

The MIC and MBC values of *P. betle* extract against the clinical isolates were determined using a broth microdilution assay. As shown in Table-3, the extract demonstrated strong anti-staphylococcal activity against *S. aureus* and *S. haemolyticus*, with similar MIC and MBC values ranging from 0.03 to 0.125 mg/mL. Furthermore, the extract showed MIC and MBC values against *S. chromogenes* of 0.5–1 mg/mL and 0.5–2 mg/mL, respectively.

**Table-3:** The MIC and MBC values of *Piper betle* extract against clinical isolates of staphylococci isolated from bovine mastitis.

Clinical isolates	MIC/MBC (mg/mL)	

	*Piper betle*extract	Vancomycin
*Staphylococcus aureus*		
WU211001	0.06/0.06	0.001/0.001
WU211002	0.125/0.125	0.001/0.001
WU211003	0.06/0.06	0.0005/0.0005
WU211004	0.06/0.06	0.0005/0.0005
WU211005	0.03/0.03	0.001/0.001
*Staphylococcus chromogenes*		
WU211024	0.5/1	0.002/0.002
WU211025	0.5/0.5	0.001/0.002
WU211037	1/1	0.0005/0.001
WU211038	1/1	0.0005/0.0005
WU211039	1/2	0.001/0.001
*Staphylococcus haemolyticus*		
WU211013	0.06/0.125	0.002/0.002
WU211014	0.125/0.125	0.001/0.001
WU211019	0.125/0.125	0.001/0.002
WU211028	0.125/0.125	0.002/0.002
WU211033	0.125/0.125	0.0005/0.001
*Staphylococcus aureus*		
ATCC25923	0.25/0.25	0.005/0.001

MIC=Minimum inhibitory concentration, MBC=Minimum bactericidal concentration

The antibacterial activity of BSP against bovine mastitis-inducing staphylococci was tested *in vitro*. BSP displayed antibacterial activity against the clinical isolates, with MIC and MBC values ranging from 12.5 to 50 v/v (Table-4). In contrast, the gel base solution exhibited no antibacterial activity against the isolates.

**Table-4:** Antibacterial activity of bovine teat dipping solution containing *Piper betle* extract against bovine mastitis-inducing staphylococci.

Clinical isolates	Antibacterial activity (v/v)			

	BPS		Gel base solution	
	
	MIC	MBC	MIC	MBC
*Staphylococcus aureus*WU211001	25	25	>100	>100
*Staphylococcus aureus*WU211002	25	25	>100	>100
*Staphylococcus chromogenes*WU211024	25	25	>100	>100
*Staphylococcus chromogenes*WU211025	12.5	25	>100	>100
*Staphylococcus haemolyticus*WU211013	12.5	12.5	>100	>100
*Staphylococcus haemolyticus*WU211014	25	25	>100	>100
*Staphylococcus aureus*ATCC25923	50	50	>100	>100

BPS: Bovine teat dipping solution containing *P. betle* extract MIC=Minimum inhibitory concentration, MBC=Minimum bactericidal concentration

### Antibiofilm activity

**Figure-3:**
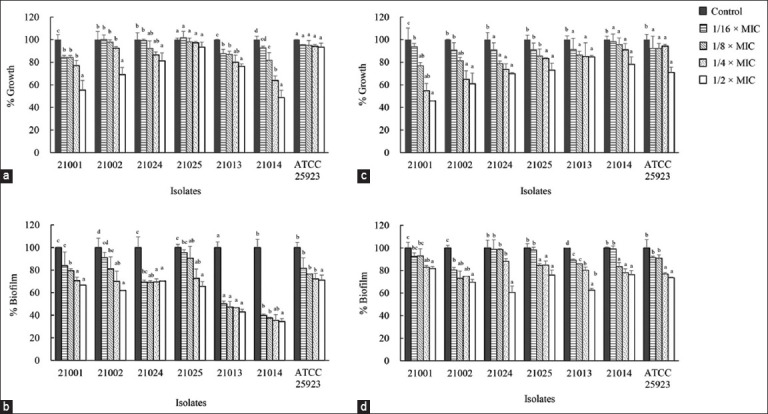
(a and b) Effects of *Piper betle* extract and (c and d) BSP on (a and c) staphylococcal growth and (b and d) biofilm formation. *Staphylococcus aureus* was WU21001 and WU21002; *Staphylococcus chromogenes* was WU21024 and WU21025; *Staphylococcus haemolyticus* was WU21013 and WU21014. Comparisons between means were determined according to *post hoc* test. Differences were considered significant at p < 0.05.

The antibiofilm activity of the extract and BSP against bovine mastitis-inducing staphylococci was assessed using a crystal violet assay. Both the extract (Figure-3b) and BSP (Figure-3d) at 1/2 and 1/4 × MIC significantly inhibited the biofilm formation of *S. aureus*, *S. haemolyticus*, and *S. chromogenes* (p < 0.05). Treatment with 1/2 × MIC of the extract and BSP resulted in biofilm inhibition ranging from 30%–66% and 19%–39%, respectively, across all isolates. However, higher plant extract concentrations inhibited the growth of certain isolates (Figures-3a and 3c).

### Elimination of the established biofilm

**Figure-4:**
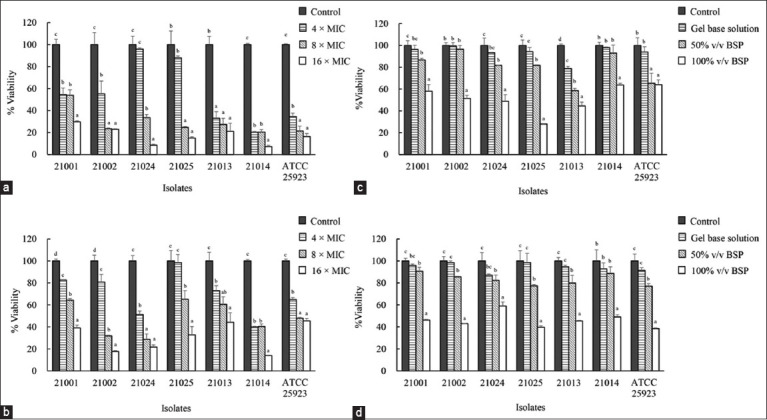
(a and b) Inhibitory activity of *Piper betle* extract and (c and d) BSP on established biofilm of staphylococci. *Staphylococcus aureus* was WU21001 and WU21002; *Staphylococcus chromogenes* was WU21024 and WU21025; *Staphylococcus haemolyticus* was WU21013 and WU21014. Comparisons between means were determined according to *post hoc* test. Differences were considered significant at p < 0.05.

The activity of the *P. betle* leaf extract against established biofilms of bovine mastitis-inducing staphylococci was evaluated using the MTT assay. Cell viability was significantly reduced both 2-day-old (Figure-4a) and 7-day-old established biofilms (Figure-4b) of *S. aureus*, *S. chromogenes*, and *S. haemolyticus* when treated with *P. betle* extract at 8 × MIC compared with the control (p < 0.05). At 16× MIC, the extract eliminated 70%–93% and 54%–86% of the established biofilm viability in the 2-day-old and 7-day-old biofilms of the staphylococcal isolates, respectively.

BSP activity eliminated established staphylococcal biofilms *in vitro*. The results revealed that treatment with 100% v/v BSP reduced 36%–72% and 41%–61% of the viability of 2-day-old (Figure-3c) and 7-day-old (Figure-3d) biofilms, respectively. In contrast, the gel base solution could not eliminate the established biofilm.

## Discussion

Staphylococci, including *S. aureus*, *S. chromogenes*, and *S. haemolyticus*, cause significant economic losses in dairy cow production by infecting and invading bovine teat cells [1]. Moreover, these bovine mastitis-inducing staphylococci can form biofilms in bovine mammary epithelial cells, which protect against pathogens in the cow’s immune system [15]. Teat disinfection before and after milking is essential for mastitis control to reduce the incidence of new intramammary infections in dairy cows. This strategy could reduce the invasion of bovine mammary cells by environmental pathogens. This study developed a bovine teat dipping solution containing *P. betle* extract as an alternative agent against bovine mastitis-inducing staphylococci.

Povidone-iodine has been commonly used as a teat dipping solution for dairy cows before and after milking because of its low toxicity and strong antibacterial effects against bovine mastitis-inducing pathogens [12]. Prepared povidone-iodine and chitosan-based carrier teat dip has also successfully prevented mastitis in dairy cows [12]. However, povidone-iodine has been found to exhibit weak antibacterial persistence when used as a medication for external applications [16]. Therefore, this study aimed to explore the properties and stabilities of BSP as a potential bovine teat dipping solution. It is important to note that BSP chemical composition is not complex, and its knowledge can be spread among farmers. The primary bioactive ingredient in BSP is *P. betle* leaf extract, a native plant in Asia, including Thailand; therefore, the application of BSP solution by farmers is possible.

*Piper betle* extract has been reported to exhibit antibacterial activity against various pathogens, including APEC [10], *Salmonella* spp. [11], extended-spectrum β-lactamase-producing *Enterobacteriaceae*, *Streptococcus* spp. [8], and methicillin-resistant *S. aureus* (MRSA) [17]. Determined MIC and MBC values in this study also indicated the potential of betel leaf extract as an antibacterial agent for bovine mastitis treatment. Moreover, the extract was considered bactericidal because the MBC/MIC ratio was < 2 [10]. The broad-spectrum antimicrobial activity of *P. betle* leaf extract makes it suitable for application as an anti-mastitis therapeutic agent, especially against environmental microorganisms that can cause bovine mastitis. A recent study by our research team revealed that hydroxychavicol and eugenol are the major phytochemicals in the ethanol extract of *P. betle* leaves [10]. Similarly, Phensri *et al*. [17] reported that these phytochemicals detected using gas chromatography-mass spectrometry analysis were the most abundant components in the ethanol betel leaf extract, which were identified as major contributors to the antibacterial properties of the betel leaves. Hydroxychavicol induces bacterial cell death via DNA damage, reactive oxygen species production, and cell division suppression [18]. Furthermore, hydroxychavicol inhibits cell division by inhibiting the formation of the FtsZ ring of *E. coli* [19]. The morphology of *E. coli* cells treated with the *P. betle* leaf extract revealed longer cells without septa compared with the control [10]. Eugenol targets the bacterial cell membrane of *S. aureus* [20]. However, inhibitory mechanisms may differ depending on the susceptibility of different bacterial species.

This study’s results indicate that both *P. betle* extract and BSP can inhibit and eliminate biofilms formed by staphylococcal isolates. A previous study [10] has reported that *P. betle* extract inhibits the APEC adhesion to surfaces, thereby inhibiting biofilm formation. In addition, the extract has been found to kill viable cells within mature biofilms and eliminate mature biofilms in APEC [10]. Eugenol, a pure compound found in *P. betle*, has been shown to inhibit cell-to-cell connections and prevents MRSA biofilm formation. Eugenol can disrupt and detach mature MRSA biofilms [21]. These findings indicate the possible mechanisms underlying biofilm inhibition and eradication by *P. betle* extract. BSP’s ability to inhibit and eliminate biofilms is considered a potential medicinal benefit in reducing mastitis persistence. Staphylococcal biofilms suppress host proinflammatory responses and macrophage phagocytosis [22]. Therefore, the immune system may remove attenuating biofilm cells from the cow’s udder. Furthermore, *P. betle* extract suppresses inducible nitric oxide synthase and cyclooxygenase-2 expression, inhibiting nitric oxide and prostaglandin E_2_ production in RAW 264.7 macrophages [23]. This indicates that the plant extract containing multiple phytochemicals may possess a wide spectrum of pharmacological effects.

To further evaluate the efficacy of BSP in preventing mastitis in dairy cows, it is recommended to conduct *in vivo* studies. These studies should assess the anti-inflammatory activity of BSP *in vitro*, clinical signs *in vivo*, and cytotoxicity. In addition, investigating the antibacterial activity of BSP against other species of bovine mastitis pathogens is important. Finally, it is advised to store BSP at low temperatures to maintain its properties as a teat dipping solution.

## Conclusion

Bovine teat dipping solution containing *P. betle* extract showed strong anti-staphylococcal activity against *S. aureus*, *S. chromogenes*, and *S. haemolyticus*. The extract’s MIC and MBC values ranged from 0.03 mg/mL to 2 mg/mL. Furthermore, BSP’s MIC and MBC values against the isolates were 12.5–50 v/v. The extract and BSP at 1/2 and 1/4 × MIC significantly inhibited staphylococcal biofilm formation. When used at 16 × MIC, the extract could eliminate 54%–86% of the established biofilm viability of 7-day-old biofilms in the isolates. BSP also inhibited 41%–61% of mature biofilm viability. Regarding BSP stability, storage at 4°C insignificantly affected the factors associated with it over 4 weeks. These results suggest that BSP derived from *P. betle* can inhibit the growth and biofilm formation of bovine mastitis-inducing staphylococci.

## Author’s Contributions

PS and WM: Conceived and designed the experiments. PS, HK, WK, SS, JCO, and WM: Performed the experiments. PS, HK, PhS, VN, MLP, and WM: Analyzed and interpreted the data. PS, PhS and WM: Performed statistical analyses. VN and MLP: Contributed to the reagents, materials, analytical tools, and data. PS, VN, MLP, and WM: Drafted the manuscript. All authors have read and approved the final manuscript.
